# Significance of mouse xenograft tumor model using patient-derived cancer organoids for clinical drug development

**DOI:** 10.3389/fonc.2025.1485886

**Published:** 2025-02-26

**Authors:** Hisataka Ogawa, Keiichi Yoshida, Shinichiro Hasegawa, Hiroshi Wada, Masayoshi Yasui, Hideaki Tahara

**Affiliations:** ^1^ Nitto joint Research Department for Nucleic Acid Medicine, Research Center, Osaka International Cancer Institute, Osaka, Japan; ^2^ Next-generation Precision Medicine Research Center, Osaka International Cancer Institute, Osaka, Japan; ^3^ Department of Gastroenterological Surgery, Osaka International Cancer Institute, Osaka, Japan; ^4^ Department of Cancer Drug Discovery and Development, Research Center, Osaka International Cancer Institute, Osaka, Japan; ^5^ Center for Clinical Research, Osaka International Cancer Institute, Osaka, Japan

**Keywords:** bile duct cancer, tumor organoid, drug screening, cell-line derived xenograft tumor model, organoid-derived xenograft tumor model

## Abstract

**Background:**

*In vitro* and *in vivo* preclinical examinations of cancer cell lines are performed to determine the effectiveness of new drugs before initiating clinical trials. However, there is often a significant disparity between the promising results observed in preclinical evaluations and actual outcomes in clinical trials. Therefore, we hypothesized that this inconsistency might be due to the differences between the characteristics of cell lines and actual cancers in patients. Therefore, we screened drugs for bile duct cancer to test our hypotheses.

**Methods:**

We established patient-derived cancer organoids (PDCOs) from the surgical samples of patients with bile duct cancer and conducted multiple *in vitro* drug screening tests.

**Results:**

We identified proteasome inhibitors (Bortezomib and Carfilzomib) as promising drugs in the screening. Bortezomib has demonstrated a significant antitumor effect on bile duct cancer cell-derived xenografts, as previously reported in preclinical trials. However, although Bortezomib showed significant proliferation inhibition in PDCOs in three-dimensional culture *in vitro*, it did not exhibit significant anti-tumor effects in mouse xenograft tumor models using our PDCOs. Bile duct cancer cell-line-derived xenografts are characterized by structurally uniform, irregular glandular structures surrounded by simple and sparse stromal components. However, organoid-derived xenografts exhibit a spectrum of differentiation levels within irregular glandular structures and consist of a complex and rich stromal microenvironment similar to those observed in surgical specimens.

**Conclusion:**

These findings suggest that *in vivo* studies using PDCO xenograft tumor models may be more suitable than conventional mouse tumor models for determining the clinical development of drugs.

## 1 Introduction

Bile duct cancer (BDC; cholangiocarcinoma) is a relatively rare gastrointestinal cancer, ranked 13th in incidence among all cancers, with poor prognosis and < 40% 5-year overall survival in Japan (1). Cholangiocarcinomas are a diverse group of malignancies that originate in the biliary system, the majority of which are adenocarcinomas. These cancers are classified based on their anatomical location, including intrahepatic cholangiocarcinoma (arising within the liver), perihilar cholangiocarcinoma (occurring at the junction of the liver and bile ducts), distal cholangiocarcinoma (affecting the bile ducts outside the liver), ampullary carcinoma, and gallbladder cancer. The management of cholangiocarcinoma, particularly perioperative care and surgical approaches for curative resection, varies considerably depending on the tumor’s location. Additionally, the biological characteristics of these tumors, such as the molecular mechanisms of carcinogenesis, histological features, and malignancy grading, can significantly differ between subtypes. These biological distinctions contribute to variability in clinical behavior, treatment responses, and prognostic outcomes. Understanding these differences is critical for developing tailored therapeutic strategies and improving patient outcomes (1).

Invasive surgery involving extensive liver and multi-organ resection remains the only curative treatment for cholangiocarcinoma. However, owing to high recurrence and metastasis rates even after curative surgery, the development of effective systemic treatments is crucial. Systemic treatment for cholangiocarcinoma is mainly limited to combinations of chemotherapeutic drugs (2, 3). Furthermore, cancer genomic medicine, which targets genetic abnormalities, has gained attention, particularly in patients with lung cancer, where it has improved patient prognosis (4). However, in patients with cholangiocarcinoma, this approach has been less successful owing to the lower number of targetable gene abnormalities, considering that the reported targetable genetic abnormalities differ based on tumor localization (5). Therefore, biological assays are useful for the development of therapeutic drugs against cholangiocarcinoma. Traditionally, cancer cell lines have been used in preclinical studies to evaluate drug potency. These conventional cell lines propagated in two-dimensional (2D) cultures have been used for large-scale *in vitro* drug screening and preclinical evaluation in mouse tumor models. However, the promising *in vivo* anti-tumor effects observed in these models often do not correlate with clinical trial outcomes for patients, especially for solid tumors. Therefore, we hypothesized that assays using patient-derived cancer organoids (PDCOs) would yield clinically relevant results. PDCOs can maintain biological characteristics such as the heterogeneity inherent in patient tumors and preserve the cancer microenvironment when xenografted *in vivo* (organoid-derived xenografts [ODX]) (6–8). Thus, in this study, we established PDCOs using surgical specimens from patients with cholangiocarcinoma treated in our facility and investigated the advantages of PDCOs in non-genetic *in vitro* drug screening and evaluation of drug susceptibility in the ODX tumor model.

## 2 Patients and methods

### 2.1 Patients and sample collection

This study was conducted in accordance with the guidelines of the Institutional Review Board for Clinical Research, and informed consent was obtained from all patients. Between January 2022 and December 2023, we established the PDCOs for cholangiocarcinoma, colorectal cancer, and duodenal cancer. Surgeons provided samples ranging in size from 2 to 3 mm to 2–3 cm. The medical records of the patients involved in this study were appropriately utilized following approval from the ethics committee of the Osaka International Cancer Institute (approval number: 23142).

### 2.2 Reagents

This work was supported by the Research Support Project for Life Science and Drug Discovery at the Drug Library. They provided a Food and Drug Administration (FDA)-approved drug library comprising 1,143 compounds in four separate 384-well plates at a concentration of 10 mM. The drug library used in this study was prepared previously and included platinum-based anticancer agents, such as cisplatin and carboplatin; however, oxaliplatin was not included. Similarly, paclitaxel and irinotecan were included, but nanoliposomal irinotecan (NAL-IRI) was not. After the first screening, the following compounds were purchased as candidate drugs: Bortezomib, Carfilzomib, Idarubicin, Doxorubicin, Epirubicin, Mitoxantrone, Daunorubicin, Camptothecin, Gemcitabine, Cisplatin, and Fluorouracil, all of which were (DMSO) (TargetMol, Boston, MA, USA).

### 2.3 Establishment of patient-derived tumor organoids and 2D culture

**Figure 1:**
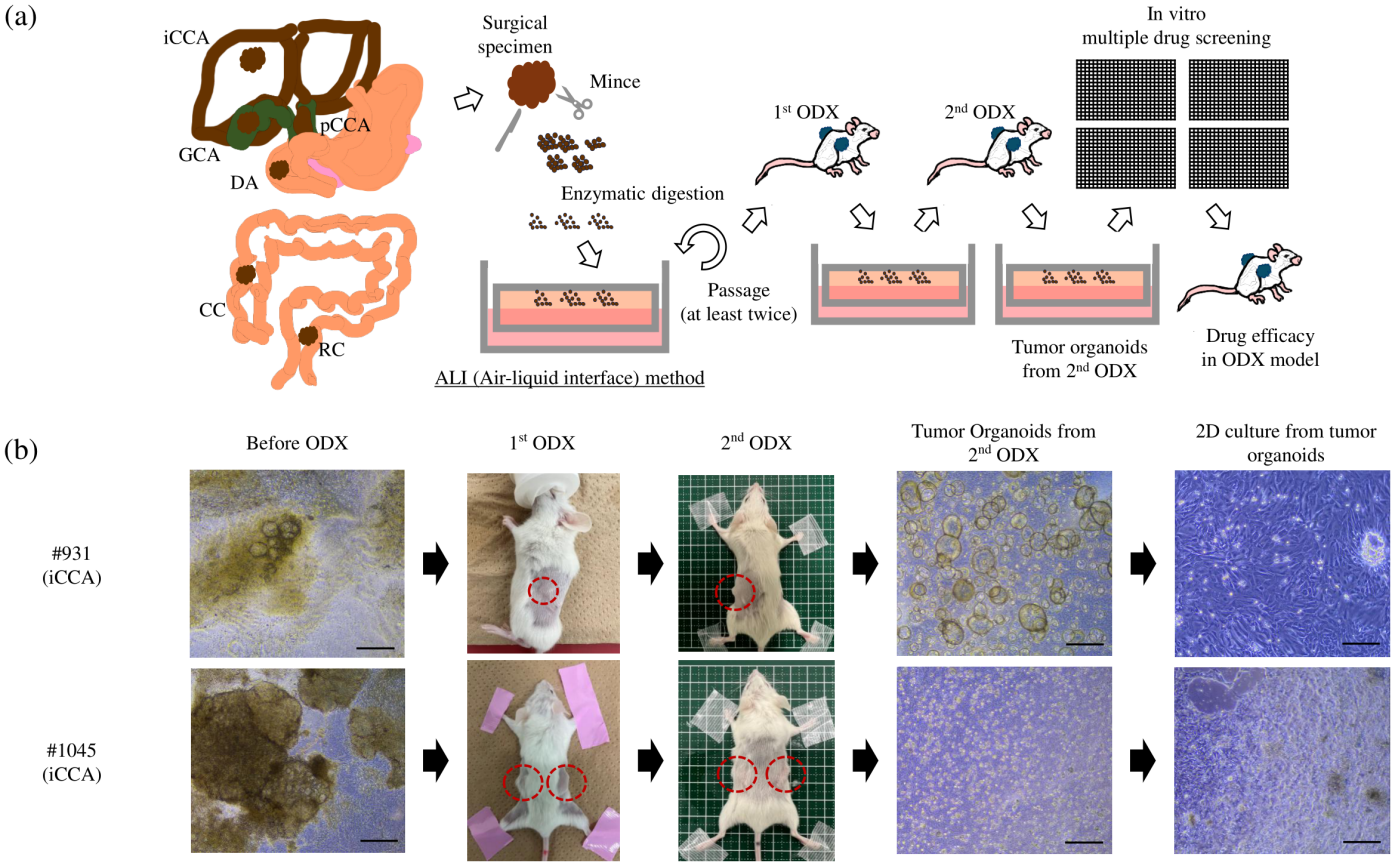
**(A)** Overview of the protocol used to establish tumor organoids from surgical samples: Surgical samples were minced, followed by enzymatic digestion into the ALI organoid culture. After at least two passages *in vitro*, the entire organoids were inoculated onto the flank of the NSG mice. After tumor formation, the tumors were removed for *in vitro* organoid culture again. This process was repeated twice, and then we defined cultured organoids from the second ODX as PDCOs. The two-dimensional culture derived from PDCO was used for drug screening, and the anti-tumor effect of the identified candidate drug was evaluated in the ODX model. **(B)** Two PDCOs (#931 and #1045 patient) were established, as mentioned in **(A)**. Scale bar-200 μm. ALI, Air-liquid interface; ODX, Organoid-derived xenograft; PDCO, Patient-derived cancer organoid; iCCA, intrahepatic cholangiocarcinoma; pCCA, perihilar cholangiocarcinoma; RC, rectal cancer; CC, colon cancer; DA, duodenal cancer; GC, gallbladder cancer; NSG, NOD.Cg-Prkdcscid Il2rgtm1Wjl/SzJ.

PDCOs were prepared using the air-liquid interface (ALI) method, as described in our previous report, which demonstrated the use of the ALI method to establish tumor organoids from various sarcomas that are challenging to cultivate using conventional 2D culture methods (9). The complete organoid medium used in this study has been described previously (10). In detail, the complete organoid medium comprised Basal medium (Advanced DMEM/F12, 1x Penicillin-Streptomycin-amphotericin-B, 1x GlutaMAX, 10 mM HEPES) (Thermo Fisher Scientific, Waltham, MA, USA) supplemented with 1x B27 supplement (Gibco, Grand Island, NY, USA), 1x N2 supplement (Thermo Fisher Scientific), 1.25 mM N-acetyl-L-cysteine (WAKO, Osaka, Japan), 1 μg/ml Recombinant Human R-Spondin-1 (R&D Systems, Minnneapolis, MN, USA), 10% Afamin/Wnt3a CM (MBL, Tokyo, Japan), 10 nM nicotinamide (Sigma Aldrich), 10 nM recombinant human (Leu15)-gastrin I (Sigma Aldrich), 50 ng/ml recombinant human EGF (Sigma Aldrich, St. Louis, MO, USA), 50 ng/ml recombinant human FGF10 (WAKO), 100 ng/ml recombinant human HGF (Sigma Aldrich), 10μM forskolin (Sigma Aldrich), 5 μM A8301 (Tocris, Avonmouth, UK), 25 ng/ml Noggin (Peprotech, Cranbury, NJ, USA), 10 μM Human Y27632 (WAKO), and 3 nM dexamethasone (Sigma Aldrich). Since this complete medium supports the growth of normal and cancer cells, after at least two passages *in vitro*, the expanded organoids were inoculated onto the flank of immunodeficient NOD.Cg-Prkdcscid Il2rgtm1Wjl/SzJ (NSG) mice for *in vivo* selection of the cancerous components. Following the first tumor formation (ODX), the tumors were harvested and re-cultured *in vitro* as organoids. This process was repeated, and the tumor organoids were cultured from the second ODX to establish PDCOs (**Figure 1A**). The propagated PDCOs were frozen in a Stem Cell Banker (Amsbio ZENOAQ, 11890, UK) and stored in a -80°C freezer. For the 2D culture, once the thawed tumor organoids began growing, they underwent trypsin treatment into single cells and were seeded on a 384-well collagen-coated culture plate (CELLCOAT Collagen μClear 384 well, Greiner, Austria) with organoid culture medium.

### 2.4 First multiple drug screening and second screening

Two-dimensional (2D) cultures derived from PDCOs were used for drug screening. We initially determined the number of plated cells based on stable cell growth using the RealTime-Glo MT Cell Viability Assay Kit (Promega, Madison, WI, USA). Single cells from two PDCOs (#931 and #1045) were plated in a 384-well collagen-coated plate in 40 μL of culture medium. After overnight culture, we added 40 μL of the culture medium containing twice the real-time cell viability reagents to each well. Luminescence values were measured using an EnSight Multimode Plate Reader (Perkin Elmer, MA, USA) for the next 3 consecutive days, including a starting point at 1 h after adding the real-time cell viability reagents. Subsequently, we determined the number of plated cells and the concentration of the solvent (DMSO) that did not affect cell proliferation in the same manner.

Furthermore, we conducted the first multiple-drug screen using a 1,134-compound FDA-approved drug library. We used a one-compound-per-well screening method to measure time-dependent changes in luminescent values. Notably, all compounds were assessed at a single dose of 1 μM, with the positive and negative controls corresponding to 6.5μM Epirubicin (TargetMol, Boston, MA, USA) (2I-2P and 23A-H in each plate) and 0.05 μM DMSO (2A-2H and 23I-P in each plate), respectively. After identifying several drugs with strong anti-cancer effects, we conducted a second screening of the identified drug candidates. The second screening was performed in triplicate at three different concentrations to identify the drug with the highest potential efficacy. For the drug screening conducted in 384-well plates, we aimed to identify compounds that demonstrated rapid and potent growth inhibition. To achieve this, we set the observation period to the shortest possible duration of three days, allowing us to evaluate the effects of drugs that act on the cell cycle. We used the RealTime-Glo reagent to monitor cell proliferation over time during the screening process. While we also considered conducting long-term drug exposure tests, we opted for a three-day observation period to minimize technical errors, such as those related to medium changes, by eliminating the need for culture medium replacement.

### 2.5 *In vitro* cell proliferation assay

A cell proliferation assay was performed to evaluate whether the drugs identified using 2D culture-based screening exhibited drug sensitivity in a 3D culture system. The efficacy of the drugs on tumor organoids in 3D culture was assessed by scaling down the ALI culture method to a 24-well format. Millicell culture plate inserts (PICM 01250; Millicell-CM, Millipore, MA, USA) were placed in 24-well culture plates (Thermo Fisher Scientific, WA, USA). The CellTiter-Glo^®^ 3D Cell Viability Assay was used to measure cell proliferation under each condition. The propagated #931, #1045, and #1074 PDCOs were cultured for three days using the ALI method, after which the organoid culture medium was replaced with fresh organoid culture medium containing the indicated drugs for three days. Cell proliferation was measured using the CellTiter-Glo^®^ 3D Cell Viability Assay. We also assessed the efficacy of drugs in 2D-cultured PDCOs. PDCOs #931, #1045, and #1074 were seeded in a 96-well collagen-coated plate overnight, and the culture medium was then replaced with fresh organoid culture medium containing the indicated drugs for 3 days. Cell proliferation was measured using the CellTiter-Glo^®^ Luminescent Cell Viability Assay.

### 2.6 Mouse xenograft study

In the mouse experiments, we primarily aimed to obtain PDCOs after repeated removal of non-malignant cells through ODX formation. Initially, we attempted to culture organoids from surgical samples. However, after two rounds of ODX formation, we successfully obtained from the second ODX (**Figure 1**). Briefly, cells prepared from expanding organoids were mixed with an equal volume of growth factor-reduced Matrigel (BD Biosciences). The resulting mixture (100 μL in total) was injected into one flank of 6- to 10-week-old female NSG mice using a 26-G insulin syringe. Upon detection of palpable tumors, the xenograft tumors were removed under sterile conditions for repeated *in vitro* organoid cultures. For *in vivo* experiments with the candidate drugs, cholangiocarcinoma cell lines (TFK-1: 1.5 × 10^6^ cells/tumor, HuCCT-1: 2.0 × 10^6^ cells/tumor) and PDCOs (#1045:1.5 × 10^6^ cells/tumor, #1074: 1.0 × 10^6^ cells/tumor) were injected into both flanks of 6-week-old female NSG mice. When subcutaneous tumors reached 100-200 mm^3^, the mice were divided into treatment groups. Tumor growth was measured using digital calipers, and the estimated volume was calculated according to the following formula: Tumor volume (mm^3^) = 0.5 × length × width^2^. All animal experiments were conducted according to our institutional guidelines and approved by the Institutional Committee for the Care of Animals at the Osaka International Cancer Institute (approval number: 23022127).

### 2.7 Immunohistochemical staining

We assessed the histological similarities among the surgical specimens, cultured PDCOs, and tissue samples obtained from the second ODX. The PDCOs *in vitro* and the matched ODX were fixed in 10% neutral buffered formalin, embedded in paraffin, sectioned at 4 μm thickness, and stained with hematoxylin and eosin (H&E). Immunohistochemical staining was performed using the VECTASTAIN^®^ Elite^®^ ABC Universal PLUS Kit (VECTOR PK-8200, CA, USA) following the manufacturer’s protocol. Primary antibodies against CK7 (Cat No. 66483-1-Ig, 1:1000, Proteintech, Chicago, IL, USA), using the human pancreatic cancer cell line BXPC-3 xenograft as the CK7 positive control, and CK20 (Cat No. ab76126, 1:200, Abcam, Cambridge, UK), with the human colon cancer cell line HT29 xenograft as the CK20 positive control. We also examined the histology of TFK-1- and HuCCT-1-derived xenograft tumors and #1074 ODX using H&E staining. Images were captured using an Olympus Slide Scanner VS200 microscope (Olympus).

### 2.8 Statistical analysis

Statistical analyses were performed using Prism 9.5 (GraphPad Software Inc., San Diego, CA, USA). The p-values and applied statistical tests are provided in the figure captions.

## 3 Results

### 3.1 Establishment of patient-derived tumor organoids

**Figure 2:**
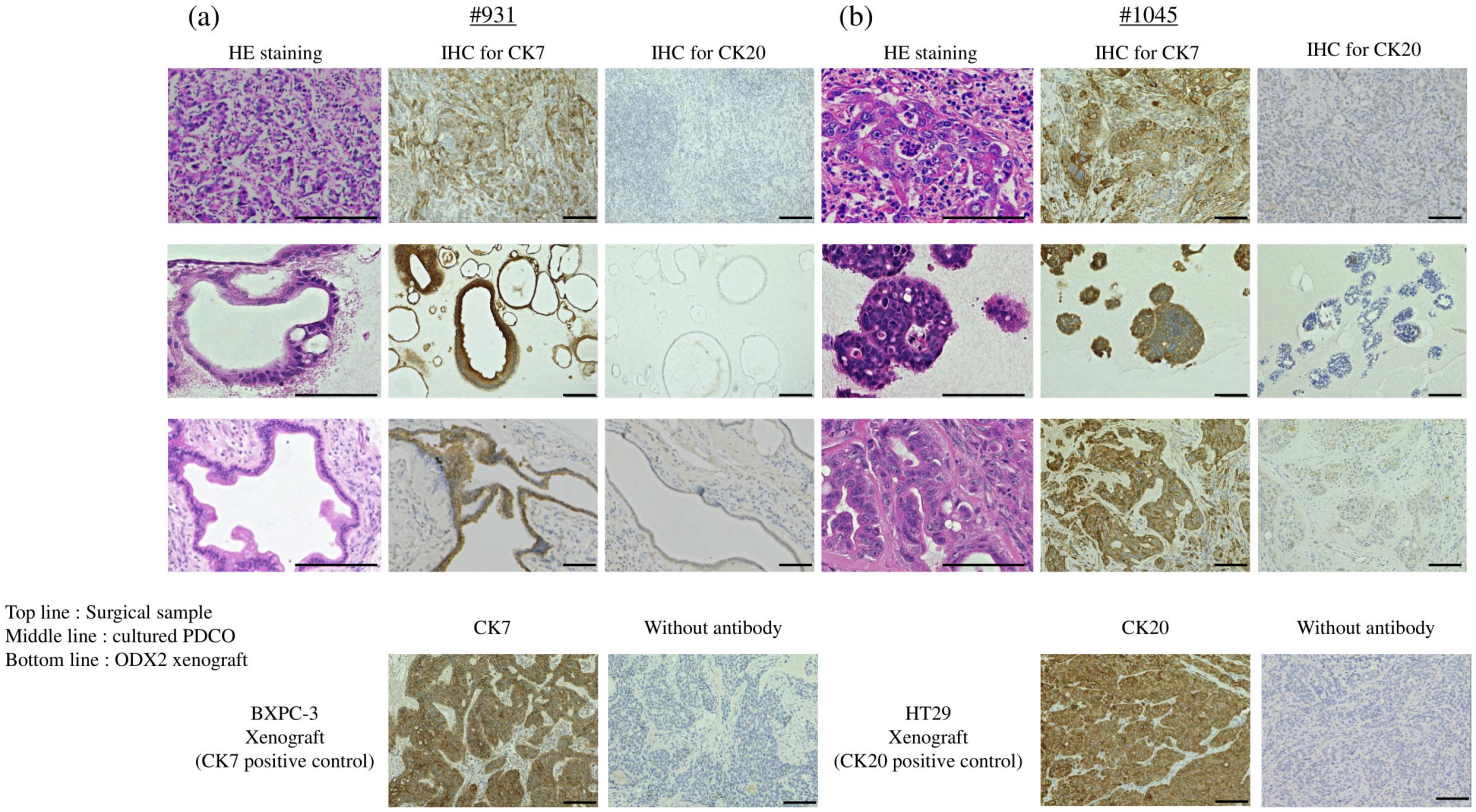
H&E staining and IHC staining in **(A)** #931 case and **(B)** #1045 case. Top line: each surgical sample, middle line: cultured PDCO, bottom line: ODX2. BXPC-3 xenograft as CK7 positive control, HT29 xenograft as CK20 positive control. Scale bar-200 μm (H&E staining) and 100 μm (CK7 and CK20 IHC staining). PDCO, Patient-derived cancer organoid; ODX, Organoid-derived xenograft; H&E, hematoxylin and eosin; IHC, immunohistochemistry. Scale bar-200 μm.

We have previously successfully established PDCOs using the ALI method in challenging cases, such as sarcomas; however, this is our first attempt to develop tumor organoids in digestive cancers. In this study, successful organoid culture was achieved in 22 patients between January 2022 and December 2023, including 19 cholangiocarcinomas (12 intrahepatic cases, including one liver metastasis, five perihilar cases, and one gallbladder case), one duodenal cancer case, and four colorectal cancer cases (**Table 1**). In the early phase of this project, we successfully established two types of PDCOs that were then used for drug screening. Notably, both cases involved patients with intrahepatic cholangiocarcinoma who received gemcitabine and cisplatin plus S-1 chemotherapy, followed by curative surgery. Histological examination after chemotherapy revealed moderately differentiated adenocarcinomas with massive necrotic areas. The organoids in both cases displayed heterogeneous morphology in culture before ODX formation. After two rounds of ODX formation, the PDCOs from the second ODX appeared homogenous (**Figure 1B**). Histopathological examination, including H&E and immunohistochemical staining for CK7 and CK20, was performed. H&E staining of PDCOs #931 and #1045 showed adenocarcinomas with atypical glandular structures, recapitulating the original primary tissue (**Figure 2**).

**Table 1:** Patients enrolled in this study.

Sample ID	Sex	Diagnosis	Preoperative Treatment	Primary culture	ODX1	ODX2
923_xx_BDC	F	iCCA	none	〇	×	
931_xx_BDC	F	iCCA	GEM+CDDP+TS1	〇	〇	〇
937_xx_BDC	F	pCCA	none	〇	In progress	
956_xx_CRC	M	RC	none	bacterial contamination	Not performed	
979_xx_CRC	F	CC	none	〇	〇	×
986_xx_CRC	M	RC	none	bacterial contamination	Not performed	
997_xx_CRC	F	CC	none	〇	In progress	
1013_xx_CRC	M	RC	none	〇	In progress	
1022_xx_BDC	M	pCCA	none	bacterial contamination	Not performed	
1025_xx_CRC	M	CC	none	〇	In progress	
1024_xx_DC	F	DA	none	〇	In progress	
1033_xx_BDC	M	pCCA	none	〇	×	
1045_xx_BDC	M	iCCA	GEM+CDDP+TS1	〇	〇	〇
1048_xx_BDC	F	liver meta from iCCA	TS1 + SBRT	〇	In progress	
1062_xx_BDC	F	pCCA	none	〇	In progress	
1074_xx_BDC	F	iCCA	none	〇	〇	〇
1136_xx_BDC	M	iCCA	none	〇	〇	In progress
1143_xx_BDC	M	iCCA	none	〇	In progress	
1197_xx_BDC	F	iCCA	none	〇	〇	In progress
1199_xx_BDC	F	pCCA	none	〇	In progress	
1209_xx_BDC	F	pCCA	none	bacterial contamination	Not performed	
1224_xx_BDC	M	iCCA	none	〇	In progress	
1221_xx_BDC	M	pCCA	none	〇	In progress	
1233_xx_BDC	M	iCCA	none	〇	In progress	
1234_xx_BDC	M	pCCA	none	bacterial contamination	Not performed	
1270_xx_BDC	F	iCCA	none	〇	In progress	
1273_xx_BDC	F	GC	GEM+CDDP+ Duligotumab	〇	In progress	
1284_xx_0809	M	BilIN	none	〇	In progress	
1358_ICC_12	M	iCCA	none	〇	In progress	

iCCA, intrahepatic cholangiocarcinoma; pCCA, perihilar cholangiocarcinoma; RC, rectal cancer; CC, colon cancer; DA, duodenal cancer; GC, gallbladder cancer; SBRT, stereotactic body radiation therapy; BilIN, biliary intraepithelial neoplasia.〇 means success ✕ means failure.

**Figure 3:**
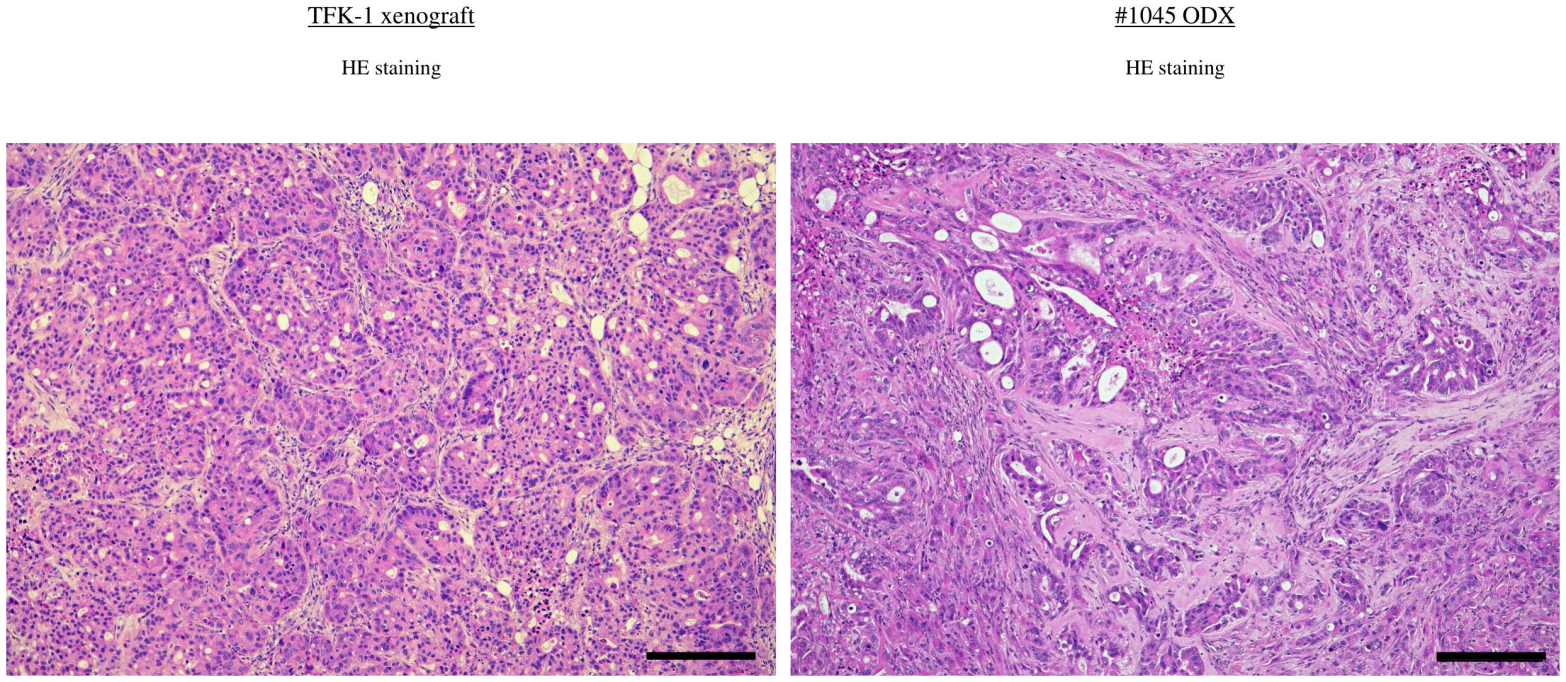
H&E staining in BDC cell line-derived xenograft (TFK-1) and ODX2 (#1045) Scale bar-200 μm. ODX, Organoid-derived xenograft; H&E, hematoxylin and eosin; BDC, bile duct cancer.

Furthermore, the ODX models exhibited histological features characterized by heterogeneous, irregular, and atypical glandular structures surrounded by complex and abundant stromal components. In comparison, TFK-1-derived xenografts showed histological features composed of uniform, irregular glandular ducts with few stromal components (**Figure 3**). For further histological investigation, we performed immunohistochemistry for CK7 and CK20 in the primary surgical specimens, PDCOs, and ODX. CK7/CK20 is typically used to identify the primary site of origin of metastatic lesions in gastrointestinal tract cancers. We observed high CK7 immunoreactivity in the cytoplasm of adenocarcinoma cells in primary tissues, PDCOs, and ODX in both cases (**Figure 2**). Furthermore, immunohistochemistry results for CK20 were negative in the primary tissues, PDCOs, and ODX in both cases (**Figure 2**). In the initial phase of this study, we successfully established three PDCOs through the second ODX purification from six patients (3/6 success rate, 50%) (**Table 1**).

### 3.2 The first multiple drug screening using 2D culture from tumor organoids

**Figure 4:**
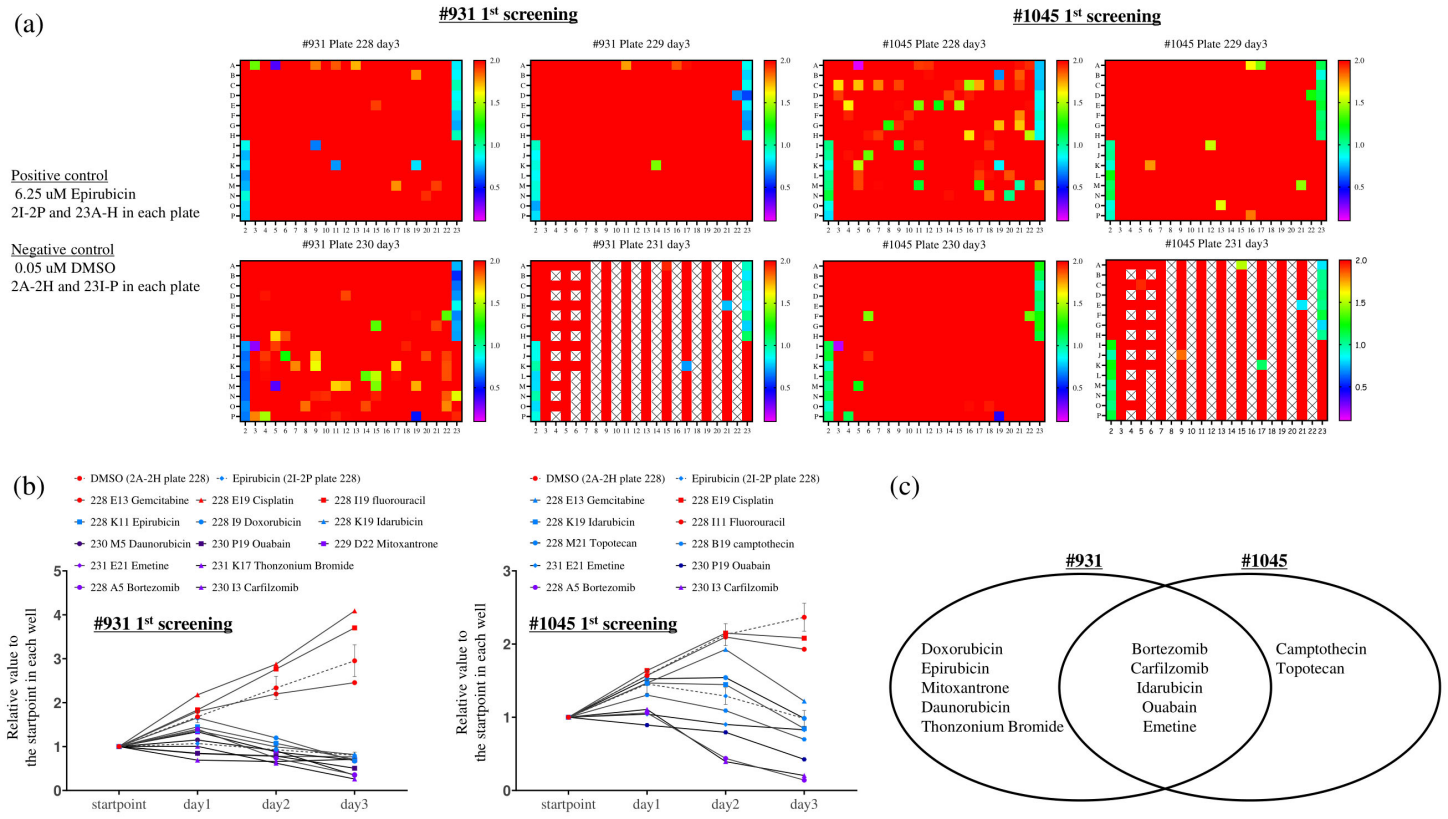
The first multiple-drug screening was conducted using a one-compound-per-well screening method with the Food Drug Administration-approved drug library (1143 compounds), which was divided into four 384-well plates. The RealTime-Glo™ MT Cell Viability Assay, capable of monitoring cellular viability over time, was employed. Cells were seeded at 250 cells/well (#931) and 125 cells/well (#1045) in 384-well plates with complete organoid culture medium overnight, and compounds were added to each well along with twice the RealTime Glo solution to achieve a concentration of 1 uM. Luminescence was measured starting from day 0 (the start point) and continued daily until day 3. **(A)** The ratio between the luminescent measurements at day 3 and day 0 (start point) was calculated, and the ratios were expressed in color-coded ranges from 0.1 to 2.0. Among these, we identified wells showing a strong growth inhibition effect, particularly those with a ratio of ≤1.0. Notably, 0.05 uM DMSO and 6.25 uM Epirubicin were used as negative (2A-2H and 23I-P in each plate) and positive controls (2I-2P and 23A-H) on each plate, respectively. X indicates blank wells. **(B)** We examined the temporal ratio of the wells identified as candidate drugs and standard chemotherapies against bile duct cancer in **(A)**. X-axis: Measurement time, Y-axis: The ratio of luminescence value at each measurement time divided by the value at each well’s start point. **(C)** Venn diagram illustrating the candidate compounds demonstrating strong growth inhibition in each case.

As the next step, we plan to conduct drug screening tests using cholangiocarcinoma PDCOs. We performed multiple drug screenings by subjecting cholangiocarcinoma PDCOs to trypsin treatment to obtain single cells and then culturing them in 384-well collagen-coated culture plates with organoid culture medium (**Figure 1B**). Notably, all three established PDCOs, including #931 and #1045, formed a monolayer and grew in 2D culture (**Figure 1B**). For multiple drug screening, we used a one-compound-per-well screening method to identify candidate drugs from an FDA-approved drug library composed of 1,134 entities in one experiment. However, to make this method reliable, it is essential to accurately measure stable cell proliferation within each well. Therefore, to achieve this, we first investigated the effect of appropriate seeding cell density and the upper limit of DMSO on cell proliferation for 3 days. These experiments indicated that the optimal plating cell number ranged from 125 to 250 cells/well in 384-well plates and that the concentration of DMSO should be < 0.5% (data not shown). We then performed the first multiple-drug screening using a 2D culture. The screening was conducted by measuring real-time cell growth at a single concentration of 1 μM for 3 days. Initially, we calculated the ratio of luminescence values on the third day of drug exposure to those on the first day in each well for all compounds. This process allowed the identification of multiple drugs that strongly inhibited cell growth (**Figure 4A**). We defined a strong inhibitory effect as a ratio < 1. This experiment followed a single compound per well” approach; therefore, it was essential to ensure the absence of technical errors during cell seeding and drug administration. To address this concern, the temporal progression of luminescence values was evaluated, and the ratio of each day to the starting point was calculated (**Figure 4B**). The analysis yielded the following results: Anthracycline anti-cancer drugs (Epirubicin, Doxorubicin), anthracenediones (Mitoxantrone, Daunorubicin), and the anti-bacterial drug (Thonzonium Bromide) showed strong inhibition only in the case of #931. However, the anti-cancer alkaloid compounds (Camptothecin, Topotecan) showed strong inhibition only in the case of #1045. Furthermore, proteasome inhibitors (Bortezomib and Carfilzomib), anthracycline anti-cancer drugs (Idarubicin), cardiac glycosides (Ouabain), and emetic compounds (Emetine) exhibited strong inhibition in both cases (**Figure 4C**). Standard chemotherapies were not effective in patient #931; however, gemcitabine showed moderate inhibition in patient #1045.

### 3.3 Second drug screening

**Figure 5:**
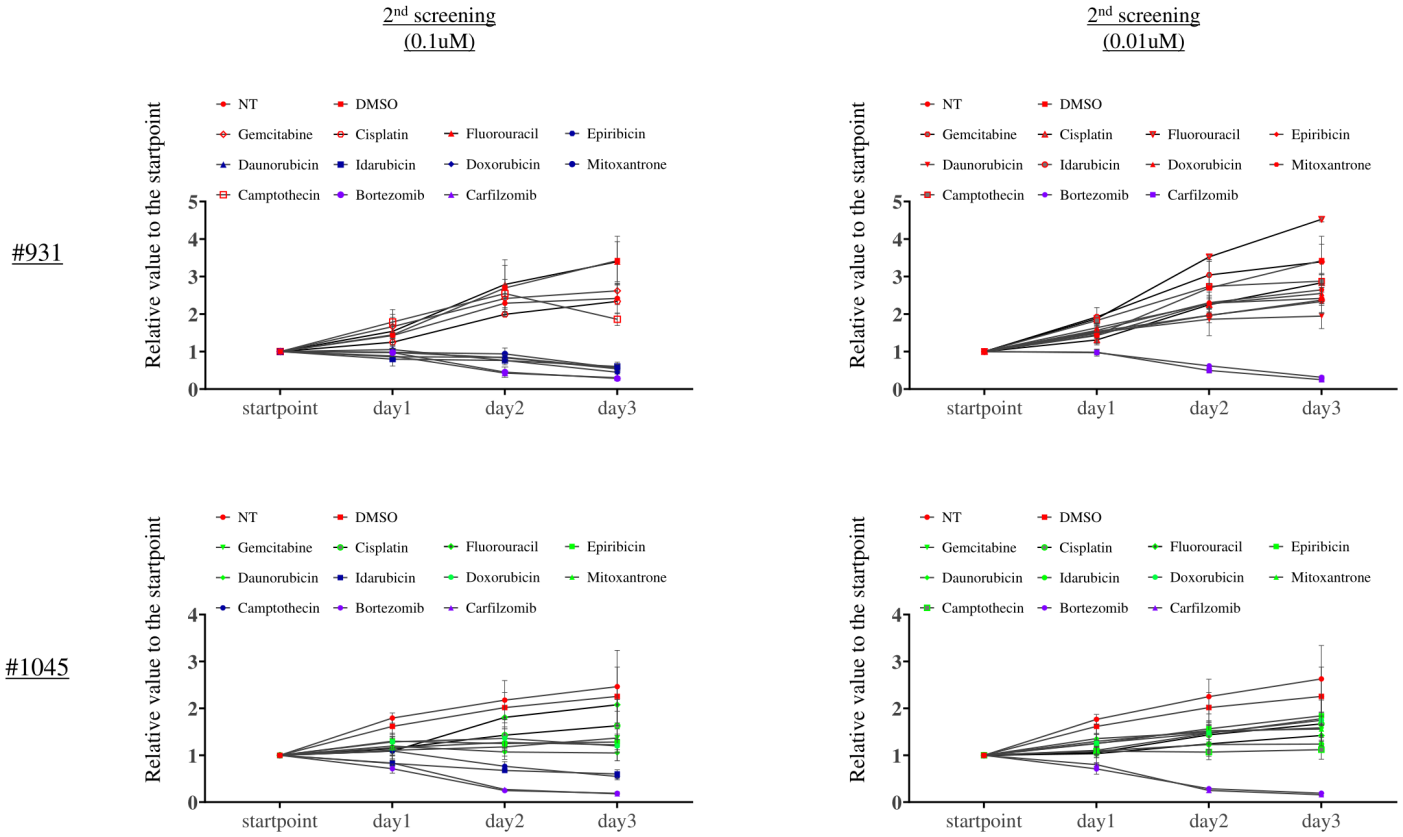
The second screening with the candidate drugs and standard chemotherapies against bile duct cancer revealed that Bortezomib and Carfilzomib were the most potent drugs. X-axis: Measurement time, Y-axis: The ratio of luminescence value at each measurement time divided by the value at each well’s start point. This assay was performed in triplicate.

We evaluated the cell growth inhibition effect of these candidate drugs (Epirubicin, Doxorubicin, Mitoxantrone, Daunorubicin, Camptothecin, Bortezomib, Carfilzomib, and Idarubicin) and standard chemotherapies against cholangiocarcinoma in triplicate in a dose-dependent manner (0.01, 0.1, and 1 μM) for 3 days. We confirmed the reproducibility between the first and second screening results at 1 μM (data not shown). Among them, only Bortezomib and Carfilzomib were the most potent even at 0.01 μM, showing a strong inhibition effect (**Figure 5**).

### 3.4 Drug sensitivity assays using the 2D and 3D culture methods

**Figure 6:**
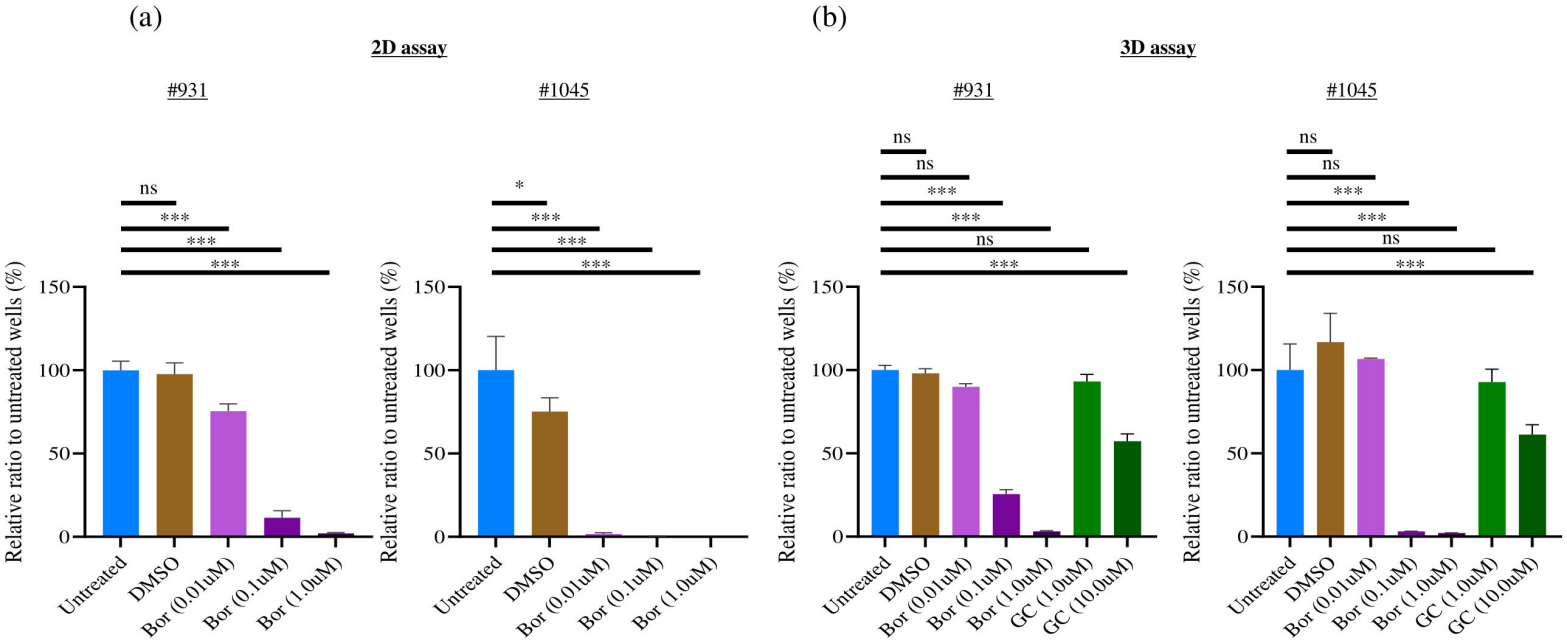
Bortezomib showed a significant strong cell proliferation inhibition towards 2D cultured PDCOs and 3D ALI PDCOs. **(A)** For the 2D assay, #1045 PDCOs were seeded at 500 cells/well in 96-well plates with complete organoid culture medium for 3 days, and compounds were added. After 3 days, the luminescence value was measured using the CellTiter-Glo^®^ Luminescent Cell Viability Assay (Promega, WI, USA). **(B)** For the 3D assay, #1045 PDCOs were seeded in a 24-well plate-based ALI organoid culture with a complete organoid culture medium for 3 days; then, the culture medium was replaced with a fresh complete organoid culture medium containing the compounds. After 3 days, the luminescence value of each well was measured using CellTiter-Glo^®^ 3D Cell Viability Assay (Promega). This assay was performed in quadruplicate. One-way ANOVA was conducted to evaluate differences among groups, followed by Bonferroni *post hoc* tests to identify specific group differences. ns, not significant,*p<0.05,*** p<0.001. 2D, two-dimensional; 3D, three-dimensional; ALI, Air-liquid interface; PDCO, Patient-derived cancer organoid.

We performed a cell proliferation assay to evaluate whether Bortezomib, a candidate drug identified from the 2D culture-based drug screening of tumor organoids, also exhibited efficacy in a 3D ALI culture system. We found that Bortezomib, at a concentration of 1 μM used in 2D culture drug screening, exhibited significant cell proliferation inhibition in the 2D culture (**Figure 6A**) and 3D ALI organoid culture system (**Figure 6B**) in both #931 and #1045 PDCOs. Similar inhibition was observed at a lower concentration of 0.1 μM. However, when the concentration was reduced to 0.01 μM, the proliferation inhibition observed in 2D cultures was not evident in the 3D ALI organoid culture system in #1045 PDCO. Although a statistically significant difference was observed with 0.01 µM Bortezomib both in the 2D and 3D culture in the #931 PDCO, the proliferation inhibitory effect was lower in 3D culture than in 2D culture (**Figure 6**). Conversely, the standard chemotherapies of Gemcitabine and Cisplatin (GC regimen) showed no significant growth inhibition at the 1 µM concentration but exhibited significant growth inhibition at the 10 µM concentration in 3D culture in both #931 and #1045 PDCOs (**Figure 6B**).

### 3.5 Mouse experiment

**Figure 7:**
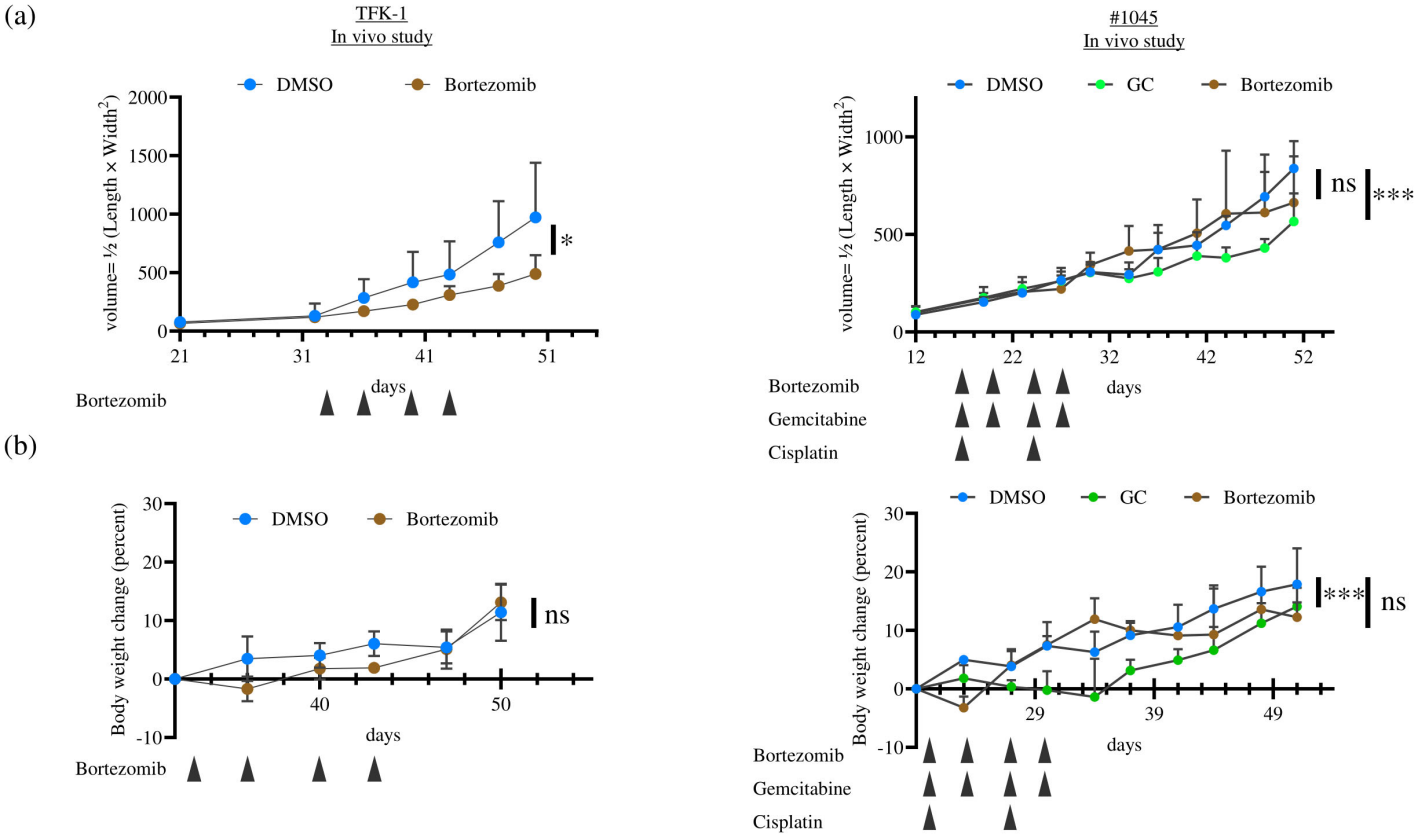
The anti-tumor effect of Bortezomib in TFK-1 xenograft tumor and ODX models was assessed for **(A)** tumor growth and **(B)** body weight change. In each experiment, TFK-1 cells or #1045 PDCOs were inoculated subcutaneously on both flanks of NSG mice. When the tumors reached 100–200 mm^3^, the mice were divided into two groups: the DMSO-treated group (5% DMSO in saline, 500 μL/mouse, intraperitoneally, twice weekly, four times in total), the Bortezomib-treated group (1 mg/kg, 500 μL/mouse, intraperitoneally, twice weekly, four times in total), or the GC-treated group (only in the #1045 ODX model, Gemcitabine 100 mg/kg, twice a week, 4 times in total, Cisplatin 4 mg/kg, once a week, twice in total) (n = 6 for each). Tumor volume was calculated using the following formula: 0.5 × length × width^2^. The results compared with the DMSO-treated group were analyzed using a two-way ANOVA with Bonferroni or Tukey’s *post hoc* test. ns, not significant, * p<0.05, *** p<0.001 TFK-1, bile duct cancer cell line; ODX, Organoid-derived xenograft; PDCO, Patient-derived cancer organoid; NSG, NOD; Cg-Prkdcscid Il2rgtm1Wjl/SzJ; DMSO, dimethyl sulfoxide.

Based on the *in vitro* results, we performed an animal experiment to evaluate the *in vivo* anti-tumor effect of Bortezomib in the ODX model compared with that in the cell line-derived xenograft tumor model. TFK-1 and #1045 PDCOs propagated *in vitro* were inoculated subcutaneously on both flanks of NSG mice. Once tumors were established and grew to 100–200 mm³, mice were divided into the vehicle control (10% DMSO in saline), Bortezomib (1 mg/kg/day, 10% DMSO in saline, twice weekly, four times in total), or GC (Gemcitabine 100 mg/kg, twice a week, four times in total; Cisplatin 4 mg/kg, once a week, twice in total) group (only in the #1045 ODX model). The dosage of these drugs was determined based on previous *in vivo* mouse experiments utilizing the maximum tolerated dose in mice (11, 12). Bortezomib treatment significantly inhibited tumor growth in TFK-1 xenograft tumor models, as previously reported (11). In contrast to the strong inhibition of cell growth observed *in vitro*, Bortezomib showed no anti-tumor effect in the #1045 ODX model (**Figure 7A**). In contrast, the GC regimen, the standard chemotherapeutic approach for cholangiocarcinoma, exhibited significant antitumor effects in the #1045 ODX model (**Figure 7A**). Mice treated with Bortezomib transiently lost body weight; however, they regained body weight by the end of the experiments under the treatment protocol (**Figure 7B**) (11). To strengthen our hypothesis, we conducted a similar experiment using another cholangiocarcinoma cell line (HuCCT-1)-derived xenograft tumor model, as well as an additional ODX model. Initially, we used the #931 ODX model for tumor growth inhibition experiments. However, owing to the significant variability in tumor growth among mice, we opted to use the #1074 ODX model, which demonstrated more consistent tumor growth. In the HuCCT-1 xenograft tumor model, we observed a significant anti-tumor effect of Bortezomib, similar to that observed in the TFK-1 xenograft tumor model (**Supplementary Figure S1A**). The cholangiocarcinoma cell lines TFK-1 and HuCCT-1 also exhibited sensitivity to Bortezomib in 2D culture (**Supplementary Figure S2A**). The #1074 PDCO also showed similar sensitivity to Bortezomib in 2D culture and to both Bortezomib and GC in 3D culture as #1045 (**Supplementary Figure S2B**). Conversely, although standard GC therapy exhibited a significant anti-tumor effect in the #1074 ODX model, Bortezomib did not demonstrate any anti-tumor activity (**Supplementary Figure S1A**), whereas #1074 PDCO showed sensitivity to Bortezomib in both 2D and 3D cultures (**Supplementary Figure S2B**). Mice treated with Bortezomib in both HuCCT-1 and #1074 ODX models showed body weight changes (**Supplementary Figure S1B**). These *in vivo* results were consistent with those obtained from the TFK-1 xenograft tumor model and the #1045 ODX model (**Figure 7**). Additionally, HuCCT-1-derived xenografts showed similar histological features, comprising uniform irregular glandular ducts with scarce stromal components as in TFK-1 xenograft tumors (**Supplementary Figure S3**). Additionally, histological examination revealed that the #1074 ODX tumor exhibited a tissue morphology similar to that of #1045, characterized by heterogeneous, irregularly shaped, atypical glandular structures surrounded by abundant stroma. (**Supplementary Figure S3**).

## 4 Discussion

Treatment of cholangiocarcinoma requires the development of new drugs that do not target genetic abnormalities because of the limited number of reported targetable genetic mutations. However, to develop and discover new drugs, it is essential to expose actual cholangiocarcinoma cell lines to various drugs to confirm their efficacy. Consequently, assays using established cancer cell lines are often used in drug development. Promising results obtained from *in vitro* and mouse tumor models frequently fail to translate into clinical trials, leading to unsuccessful drug development. This might be because, in addition to the limited number of available established cancer cell lines, cancer cell lines lose the heterogeneity inherent in the original tumor, and cell line-derived xenografts exhibit significant histological differences from patient tumors, which are considered disadvantageous in terms of drug susceptibility (13). Therefore, as an alternative method, patient-derived xenograft models, established by directly transplanting tumor tissues into immunodeficient mice, are reported to maintain the tumor’s heterogeneity; however, they have a low success rate and require a considerable amount of time and financial resources, such as requiring many mice (14, 15). Thus, PDCOs have been considered to be a powerful tool that could replace traditional cancer cell lines in clinical drug development, since organoid cultivation, utilizing *in vitro* 3D culture methods with a culture medium suitable for stem cell growth, allows for the maintenance and cultivation of tumor “stem” cells within the tumor tissue, thereby efficiently growing *in vitro* and *in vivo* while preserving the tumor’s heterogeneity. In this study, we explored new drug candidates for cholangiocarcinoma by utilizing PDCOs, which are thought to closely mimic a patient’s tissue architecture. We also used a drug library of clinically approved drugs for other diseases to identify potential therapeutic agents for cholangiocarcinoma.

We attempted to establish tumor organoids from surgically resected cholangiocarcinoma samples at our institute. The success rate of establishing cholangiocarcinoma PDCOs using another Matrigel-embedded organoid cultivation method was reported to be approximately 40% (16). In this study, we attempted to establish PDCOs by initiating the cultivation of all constituent cell populations in patient tumor tissues using the ALI method with a complete organoid culture medium and purifying the cancerous component through two rounds of ODX formation. Notably, various cell populations within the tumor were recognizable at the start of cultivation; however, the purified tumor organoids exhibited a homogeneous cellular appearance after ODX formation (**Figure 1B**). The success rate of the PDCO establishment in this study was 50%, which is comparable to or even better than that in a previous report. The Matrigel-embedded organoid cultivation method was not implemented in this study; therefore, a comparison with the results of the ALI method could not be made. However, other reports indicate that in the Matrigel method, strong enzymatic treatment disperses cells to the single-cell level, whereas the ALI method utilizes relatively mild enzymatic treatment, preserving cell-cell adhesion and allowing the culture of the entire cell population within the tissue, including stromal cells other than cancer cells. Therefore, ALI allows better preservation of the tissue architecture found in patient tumors, making it a more favorable cultivation method for cancer tissues (17, 18).

A direct comparison of genetic mutation and expression analyses between the original surgical resection sample, ODX model, and established PDCOs was not conducted in this study; however, tumor organoids established using the ALI method have been reported to retain the genetic mutations of the original tumor (6, 9). Histological evaluation confirmed that the established PDCOs and ODXs maintained histological features similar to those of the original tumor (**Figure 2**).

We successfully established cholangiocarcinoma PDCOs and then attempted to explore the candidate drugs for cholangiocarcinoma using cholangiocarcinoma PDCOs. Therefore, we used 2D cultures derived from PDCOs for multiple *in vitro* drug screening tests. The direct use of PDCOs cultivated using ALI for drug screening is challenging. The reasons include the lack of small-scale culture equipment, such as 96-well or 384-well plates, for the ALI method, and the absence of established methods for accurately observing the simultaneous proliferation of large numbers of organoids. To overcome this difficulty, we adapted the established cholangiocarcinoma PDCOs into a simplified and uniform 2D culture system. Our present drug screen using 2D cultures derived from two types of cholangiocarcinoma PDCOs successfully identified proteasome inhibitors as the most potent drugs. The ubiquitin-proteasome system is responsible for the proteasome-mediated degradation of ubiquitin-tagged proteins and is crucial for various cellular functions (19). Therefore, it is also considered a critical mechanism for cancer cell survival. This result is consistent with previous high-throughput screening results for a large number of drugs performed against a National Cancer Institute panel of 60 human hematological and solid cancer cell lines (20). The transition from 3D to 2D organoid culture has raised significant discussion regarding its impact on the cellular phenotype of tumor organoids (21–23). Therefore, we investigated the sensitivity of Bortezomib, which was identified in a 2D culture-based screening of tumor organoids in a 3D ALI culture system. The efficacy of Bortezomib at extremely lower concentrations differed between the 2D and 3D ALI organoid systems; however, it demonstrated significant cell proliferation inhibition at a concentration of 1 μM, which is used in 2D culture drug screening and 3D ALI organoid cultures (**Figure 6**). Finally, we assessed drug susceptibility *in vivo* using the ODX model to evaluate whether it could predict efficacy in clinical trials. Bortezomib and Carfilzomib are currently clinically applied in multiple myeloma owing to their efficacious anti-cancer effects *in vitro* and in multiple myeloma cell line-derived xenografts (24, 25). Similar to multiple myeloma, preclinical trials using cancer cell line-derived xenograft tumor models have demonstrated significant anti-tumor effects of Bortezomib in various types of solid tumors, widely prompting phase 2 clinical trials, including those of biliary tract cancer cases (11, 26). However, these clinical trials failed with no observed anti-tumor effects (11, 27, 28). In this study, we evaluated the anti-tumor effects of Bortezomib monotherapy in TFK-1 and HuCCT-1-derived xenograft tumors and in #1045 and #1074 ODX models by administering the maximum tolerated dose in mice (29). Bortezomib demonstrated significant anti-tumor efficacy in TFK-1- and HuCCT-1-derived xenograft tumor models, which is consistent with previous reports (11, 26). However, it showed no anti-tumor effect in either ODX model despite temporary weight loss, which is considered a side effect of Bortezomib (**Figure 7**; **Supplementary Figure S1**). This result aligns with clinical trial outcomes in cholangiocarcinoma cases (30). Therefore, our results suggest that tumor organoids are comparable to cancer cell lines in *in vitro* assays. Notably, the use of tumor organoids lies particularly in the evaluation of drug efficacy in ODX mouse tumor models, highlighting their significance in drug discovery for cancer therapeutics. Generally, the drug sensitivity of cancer cells in the ODX model forming luminal structures in abundant stroma might be affected by reduced drug penetration and increased expression of drug-resistant genes induced by hypoxic conditions in the central regions of the tumor (31, 32). Furthermore, the sensitivity of cancer cells to various drugs is believed to be strongly influenced by both endogenous and exogenous factors. Endogenous factors include genetic abnormalities in cancer cells, activity of metabolic enzymes, DNA repair capacity, abnormalities in apoptotic pathways, and cell cycle irregularities. Exogenous factors, on the other hand, encompass the influence of the tumor microenvironment, including cancer stroma, hypoxic conditions, and nutrient deprivation, among several other interconnected factors. Although not investigated in this study, previous research conducted by our group on synovial sarcoma has reported that the gene expression patterns of ODX tumors are similar to those of patient tumors (9). Moreover, the gene expression profile of organoids in 3D culture retains similarity to patient tumors (6). Considering the above, it can be inferred that for drugs whose efficacy is mainly related to endogenous factors (such as gemcitabine and cisplatin evaluated in this study), *in vitro* drug efficacy (**Figure 6**) is likely to be reflected in *in vivo* experiments (**Figure 7**; **Supplementary Figures S1**, **S2B**). Conversely, for drugs that are strongly influenced by exogenous factors, the presence of the tumor microenvironment is expected to affect drug efficacy. Although we did not analyze the differences in tumor microenvironments between cholangiocarcinoma cell line-derived xenograft tumors and ODX models, the efficacy of Bortezomib evaluated in this study indicated that the presence of cancer stroma in the tumor microenvironment affects drug efficacy, as reported previously (33). Thus, the lack of Bortezomib efficacy observed in the ODX model compared to that in cholangiocarcinoma cell line-derived xenograft tumors may be attributed to these tumor microenvironmental factors. Therefore, it might be useful to consider ODX models for evaluating drug susceptibility because cell-extrinsic factors in the tumor microenvironment attenuate therapeutic efficacy. Notably, ODX models displayed heterogeneous atypical glandular structures with complicated abundant stroma, similar to the patient samples, compared to cholangiocarcinoma cell line-derived xenograft tumors with homogenous atypical glandular structures and scarce stroma (**Figure 3**; **Supplementary Figure S3**).

The present study had some limitations. Our data suggest the potential practicality of the ODX model in predicting drug susceptibility in patients, referring to the lack of efficacy observed in the ODX model, such as the failure of clinical trials of Bortezomib for cholangiocarcinoma. However, we did not thoroughly investigate the underlying mechanism of Bortezomib resistance in our cholangiocarcinoma ODX model focusing on the tumor microenvironment. Unlike Bortezomib, clinical trials of Carfilzomib for bile duct cancer have not been conducted. Therefore, considering the superiority of the ODX model in predicting drug efficacy against cholangiocarcinoma cell-line-derived xenografts, we did not perform *in vivo* efficacy experiments. However, Carfilzomib can irreversibly inhibit the proteasome, suggesting that its duration of effect may be longer than that of Bortezomib, and its superiority over Bortezomib has been demonstrated in multiple myeloma. Owing to its irreversible proteasome inhibition, Carfilzomib may show efficacy in bile duct cancer, unlike Bortezomib, which is considered ineffective owing to its failure to act through cell-extrinsic factors. In the future research, if the antitumor effects of Carfilzomib are recognized in the ODX model for cholangiocarcinoma, it could become a candidate for a new drug for clinical trials. Furthermore, it remains unclear whether the findings obtained in this study are specific to Bortezomib or whether they can be applied to other non-genetic drugs, especially those for which drug sensitivity can be affected by cell-extrinsic factors in the tumor microenvironment. Therefore, further investigations using other non-genetic drugs in our ODX model are required.

In conclusion, the results of this study emphasized the potential utility of ODX models as valuable tools for clinical drug development, offering advantages over traditional cancer cell line-derived xenograft tumor models and *in vivo* drug sensitivity tests using cancer organoids. Further investigations and refinements in the use of patient-derived tumor organoids may contribute to more accurate preclinical drug screening and help bridge the gap between preclinical results and clinical outcomes.

## Data Availability

The raw data supporting the conclusions of this article will be made available by the authors, without undue reservation.

## Supplementary material

Supplementary Figure 1 The anti-tumor effect of Bortezomib in HuCCT-1 xenograft tumors and #1074 ODX models was assessed for **(A)** tumor growth and **(B)** body weight change. In each experiment, HuCCT-1 cells or #1074 PDCOs were inoculated subcutaneously on both flanks of NSG mice. When the tumors reached 100–200 mm^3^, the mice were divided into two groups: the DMSO-treated group (5% DMSO in saline, 500 μL/mouse, intraperitoneally, twice weekly, four times in total) and the Bortezomib-treated group (1 mg/kg, 500 μL/mouse, intraperitoneally, twice weekly, four times in total) or GC-treated group (only in the #1074 ODX model, Gemcitabine 100 mg/kg, twice a week, 4 times in total, Cisplatin 4 mg/kg, once a week, twice in total) (n = 6 for each). Tumor volume was calculated using the following formula: 0.5 × length × width^2^. The results compared with the DMSO-treated group were analyzed using a two-way ANOVA with Bonferroni or Tukey’s *post hoc* test. ns; not significant, * p<0.05, *** p<0.001 HuCCT-1, bile duct cancer cell line; ODX, Organoid-derived xenograft; PDCO, Patient-derived cancer organoid; NSG, NOD; Cg-Prkdcscid Il2rgtm1Wjl/SzJ; DMSO, dimethyl sulfoxide.

Supplementary Figure 2 Bortezomib showed a significant, strong cell proliferation inhibition toward cholangiocarcinoma cell lines and 2D-cultured PDCOs and 3D ALI PDCOs. **(A)** TFK-1 and HuCCT-1 cells were seeded at 5000 cells/well in 96-well plates overnight, and compounds were added. After 3 days, the luminescence value was measured using the CellTiter-Glo^®^ Luminescent Cell Viability Assay (Promega, WI, USA). This assay was performed in quadruplicate. **(B)** For the 2D assay, #1074 PDCOs were seeded at 500 cells/well in 96-well plates with complete organoid culture medium for 3 days, and compounds were added. After 3 days, the luminescence value was measured using the CellTiter-Glo^®^ Luminescent Cell Viability Assay (Promega, WI, USA). This assay was performed in quadruplicate. For the 3D assay, #1045 PDCOs were seeded in a 24-well plate-based ALI organoid culture with a complete organoid culture medium for 3 days; then, the culture medium was replaced with a fresh complete organoid culture medium containing the compounds. After 3 days, the luminescence value of each well was measured using the CellTiter-Glo^®^ 3D Cell Viability Assay (Promega). This assay was performed in triplicate. One-way ANOVA was conducted to evaluate differences among groups, followed by Bonferroni *post hoc* tests to identify specific group differences. ns; not significant, *p<0.05,*** p<0.001. 2D, two-dimensional; 3D, three-dimensional; ALI, Air-liquid interface; PDCO, Patient-derived cancer organoid.

Supplementary Figure 3 H&E staining in BDC cell line (HuCCT-1)-derived xenograft and ODX2 (#1074) Scale bar-200 μm. ODX, Organoid-derived xenograft; H&E, hematoxylin and eosin; BDC, bile duct cancer.

## References

[1] Miyakawa S Ishihara S Horiguchi A Takada T Miyazaki M Nagakawa T. Biliary tract cancer treatment: 5,584 results from the Biliary Tract Cancer Statistics Registry from 1998 to 2004 in Japan. J Hepato-Bil Pancreat Surg. (2009) 16:1–7. doi: 10.1007/s00534-008-0015-0 19110652

[2] Sakai D Kanai M Kobayashi S Eguchi H Baba H Seo S. Randomized phase III study of gemcitabine, cisplatin plus S-1 (GCS) versus gemcitabine, cisplatin (GC) for advanced biliary tract cancer (KHBO1401-MITSUBA). Ann Oncol. (2018) 29:vii205. doi: 10.1093/annonc/mdy282 PMC10086809 35900311

[3] Ioka T Kanai M Kobayashi S Sakai D Eguchi H Baba H. Randomized phase III study of gemcitabine, cisplatin plus S-1 versus gemcitabine, cisplatin for advanced biliary tract cancer (KHBO1401- Mitsuba). J Hepato-Bil Pancreat Sci. (2023) 30:102–10. doi: 10.1002/jhbp.1219 PMC10086809 35900311

[4] Ohashi K Matsumoto S Yoh K Ohe Y Shimokawaji T Kataoka Y. Contribution to the development of precision medicine and clinical utility of nationwide lung cancer genomic screening in Japan (LC-SCRUM-Japan). J Clin Oncol. (2017) 35:e20659. doi: 10.1200/JCO.2017.35.15_suppl.e20659

[5] Morizane C Komatsu Y Takahashi H Ueno M Furuse J Kudo T. The nationwide cancer genome screening project in Japan, SCRUM Japan GISCREEN: Efficient identification of cancer genome alterations in advanced biliary tract cancer. Ann Oncol. (2018) 29:viii208–9. doi: 10.1093/annonc/mdy282.007

[6] Neal JT Li X Zhu J Giangarra V Grzeskowiak CL Ju J. Organoid modeling of the tumor immune microenvironment. Cell. (2018) 175:1972–1988.e16. doi: 10.1016/j.cell.2018.11.021 30550791 PMC6656687

[7] Vlachogiannis G Hedayat S Vatsiou A Jamin Y Fernández-Mateos J Khan K. Patient-derived organoids model treatment response of metastatic gastrointestinal cancers. Science. (2018) 359:920–6. doi: 10.1126/science.aao2774 PMC6112415 29472484

[8] Jian M Ren L He G Lin Q Tang W Chen Y. A novel patient-derived organoids-based xenografts model for preclinical drug response testing in patients with colorectal liver metastases. J Transl Med. (2020) 18:234. doi: 10.1186/s12967-020-02407-8 32532289 PMC7291745

[9] Wakamatsu T Ogawa H Yoshida K Matsuoka Y Shizuma K Imura Y. Establishment of organoids from human epithelioid sarcoma with the air-liquid interface organoid cultures. Front Oncol. (2022) 12:893592. doi: 10.3389/fonc.2022.893592 35677170 PMC9169059

[10] Broutier L Mastrogiovanni G Verstegen MM Francies HE Gavarró LM Bradshaw CR. Human primary liver cancer-derived organoid cultures for disease modeling and drug screening. Nat Med. (2017) 23:1424–35. doi: 10.1038/nm.4438 PMC5722201 29131160

[11] Vaeteewoottacharn K Kariya R Matsuda K Taura M Wongkham C Wongkham S. Perturbation of proteasome function by bortezomib leading to ER stress-induced apoptotic cell death in cholangiocarcinoma. J Cancer Res Clin Oncol. (2013) 139:1551–62. doi: 10.1007/s00432-013-1473-6 PMC11824760 23877657

[12] Peters GJ Bergman AM Ruiz van Haperen VW Veerman G Kuiper CM Braakhuis BJ. Interaction between cisplatin and gemcitabine *in vitro* and *in vivo* . Semin Oncol. (1995) 22:72–9. 7481849

[13] Francies HE Barthorpe A McLaren-Douglas A Barendt WJ Garnett MJ. Drug sensitivity assays of human cancer organoid cultures. Methods Mol Biol. (2019) 1576:339–51. doi: 10.1007/7651_2016_10 PMC6527507 27628132

[14] Vaeteewoottacharn K Pairojkul C Kariya R Muisuk K Imtawil K Chamgramol Y. Establishment of highly transplantable cholangiocarcinoma cell lines from a patient-derived xenograft mouse model. Cells. (2019) 8:496. doi: 10.3390/cells8050496 31126020 PMC6562875

[15] Leiting JL Murphy SJ Bergquist JR Hernandez MC Ivanics T Abdelrahman AM. Biliary tract cancer patient-derived xenografts: Surgeon impact on individualized medicine. JHEP Rep. (2020) 2:100068. doi: 10.1016/j.jhepr.2020.100068 32181445 PMC7066236

[16] Saito Y Muramatsu T Kanai Y Ojima H Sukeda A Hiraoka N. Establishment of patient-derived organoids and drug screening for biliary tract carcinoma. Cell Rep. (2019) 27:1265–1276.e4. doi: 10.1016/j.celrep.2019.03.088 31018139

[17] de Visser KE Joyce JA. The evolving tumor microenvironment: From cancer initiation to metastatic outgrowth. Cancer Cell. (2023) 41:374–403. doi: 10.1016/j.ccell.2023.02.016 36917948

[18] Li X Ootani A Kuo C. An air-liquid interface culture system for 3D organoid culture of diverse primary gastrointestinal tissues. Methods Mol Biol. (2016) 1422:33–40. doi: 10.1007/978-1-4939-3603-8_4 27246020

[19] Park J Cho J Song EJ. Ubiquitin–proteasome system (UPS) as a target for anticancer treatment. Arch Pharm Res. (2020) 43:1144–61. doi: 10.1007/s12272-020-01281-8 PMC7651821 33165832

[20] Monks A Scudiero D Skehan P Shoemaker R Paull K Vistica D. Feasibility of a high-flux anticancer drug screen using a diverse panel of cultured human tumor cell lines. J Natl Cancer Inst. (1991) 83:757–66. doi: 10.1093/jnci/83.11.757 2041050

[21] Muguruma M Teraoka S Miyahara K Ueda A Asaoka M Okazaki M. Differences in drug sensitivity between two-dimensional and three-dimensional culture systems in triple-negative breast cancer cell lines. Biochem Biophys Res Commun. (2020) 533:268–74. doi: 10.1016/j.bbrc.2020.08.075 32958246

[22] Howes AL Richardson RD Finlay D Vuori K. 3-Dimensional culture systems for anti-cancer compound profiling and high-throughput screening reveal increases in EGFR inhibitor-mediated cytotoxicity compared to monolayer culture systems. PloS One. (2014) 9:e108283. doi: 10.1371/journal.pone.0108283 25247711 PMC4172726

[23] Gassl V Aberle MR Boonen B Vaes RDW Olde Damink SWM Rensen SS. Chemosensitivity of 3D pancreatic cancer organoids is not affected by transformation to 2D culture or switch to physiological culture medium. Cancers (Basel). (2022) 14:5617. doi: 10.3390/cancers14225617 36428711 PMC9688175

[24] Deshantri AK Fens MHAM Ruiter RWJ Metselaar JM Storm G Mandhane SN. Complete tumor regression by liposomal bortezomib in a humanized mouse model of multiple myeloma. Hemasphere. (2020) 4:e463. doi: 10.1097/HS9.0000000000000463 32923984 PMC7455224

[25] Cengiz Seval G Beksac M. The safety of bortezomib for the treatment of multiple myeloma. Expert Opin Drug Saf. (2018) 17:953–62. doi: 10.1080/14740338.2018.1513487 30118610

[26] Zhu M. Inhibitory effects of bortezomib in a subcutaneous tumor model of H22 mouse hepatocarcinoma cells. Pathol Res Pract. (2019) 215:152388. doi: 10.1016/j.prp.2019.03.017 30914235

[27] Wang H Cao Q Dudek AZ. Phase II study of panobinostat and bortezomib in patients with pancreatic cancer progressing on gemcitabine-based therapy. Anticancer Res. (2012) 32:1027–31. 22399627

[28] Huang Z Wu Y Zhou X Xu J Zhu W Shu Y. Efficacy of therapy with bortezomib in solid tumors: A review based on 32 clinical trials. Future Oncol. (2014) 10:1795–807. doi: 10.2217/fon.14.30 25303058

[29] Kawabata S Gills JJ Mercado-Matos JR Lopiccolo J Wilson W 3rd Hollander MC. Synergistic effects of nelfinavir and bortezomib on proteotoxic death of NSCLC and multiple myeloma cells. Cell Death Dis. (2012) 3:e353. doi: 10.1038/cddis.2012.87 22825471 PMC3406586

[30] Denlinger CS Meropol NJ Li T Lewis NL Engstrom PF Weiner LM. A phase II trial of the proteasome inhibitor bortezomib in patients with advanced biliary tract cancers. Clin Colorectal Cancer. (2014) 13:81–6. doi: 10.1016/j.clcc.2013.12.005 PMC4189831 24512954

[31] Hubert CG Rivera M Spangler LC Wu Q Mack SC Prager BC. A three-dimensional organoid culture system derived from human glioblastomas recapitulates the hypoxic gradients and cancer stem cell heterogeneity of tumors found *in vivo* . Cancer Res. (2016) 76:2465–77. doi: 10.1158/0008-5472.CAN-15-2402 PMC4873351 26896279

[32] Sensi F D’Angelo E Biccari A Marangio A Battisti G Crotti S. Establishment of a human 3D pancreatic adenocarcinoma model based on a patient-derived extracellular matrix scaffold. Transl Res. (2023) 253:57–67. doi: 10.1016/j.trsl.2022.08.015 36096350

[33] Li L Zhou Y Zhang Y Hu H Mao HQ Selaru FM. A combination therapy of bortezomib, CXCR4 inhibitor, and checkpoint inhibitor is effective in cholangiocarcinoma *in vivo* . iScience. (2023) 26:106095. doi: 10.1016/j.isci.2023.106095 36843847 PMC9950944

