# Supplementary figures and images for: Significance of mouse xenograft tumor model using patient-derived cancer organoids for clinical drug development
Source: Front Oncol. 2025 Feb 26;15:1485886. doi: 10.3389/fonc.2025.1485886 (PMC11896854; doi:10.3389/fonc.2025.1485886)

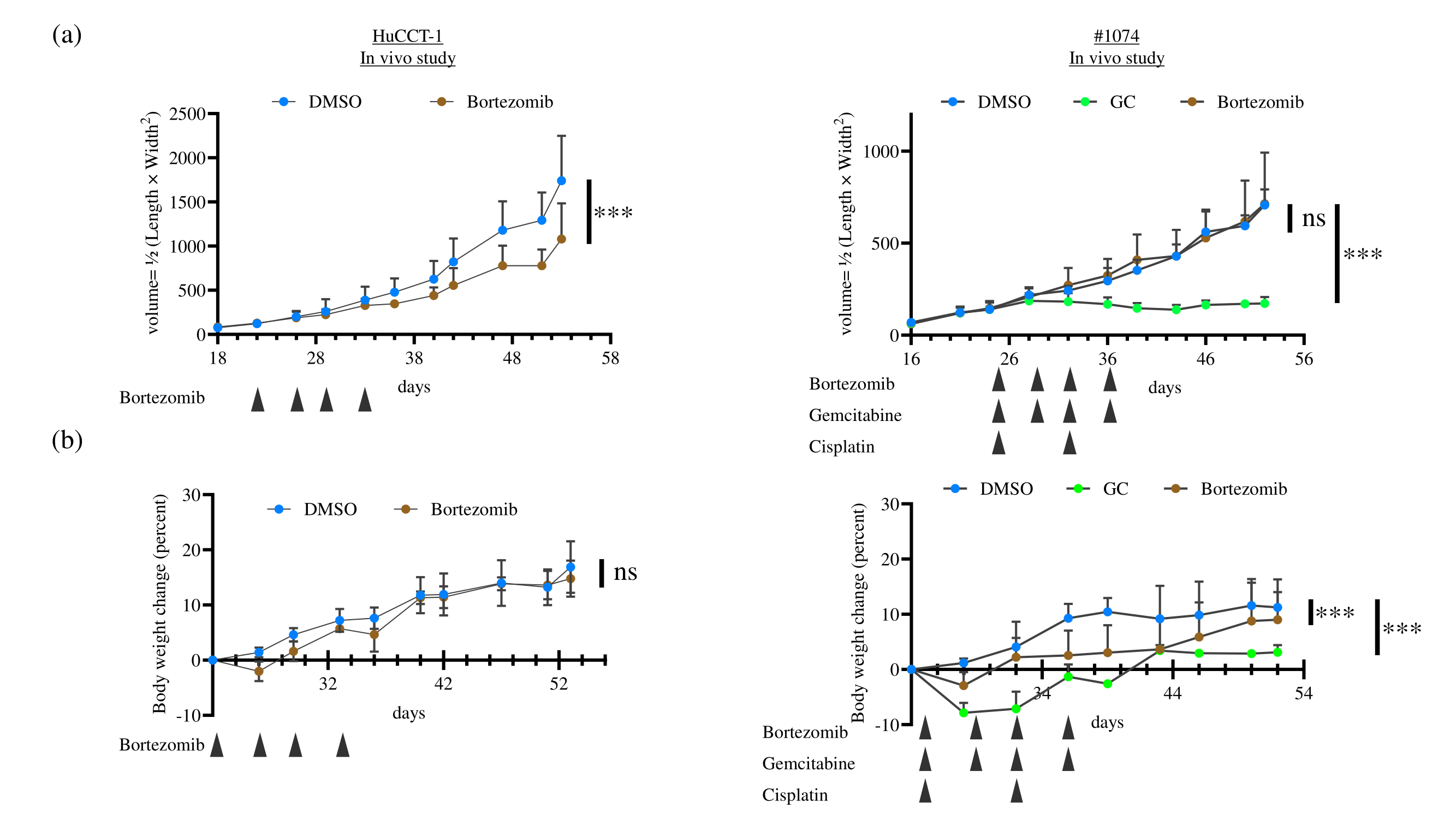

Supplement: Supplementary Figure 1 — The anti-tumor effect of Bortezomib in HuCCT-1 xenograft tumors and #1074 ODX models was assessed for (A) tumor growth and (B) body weight change. In each experiment, HuCCT-1 cells or #1074 PDCOs were inoculated subcutaneously on both flanks of NSG mice. When the tumors reached 100–200 mm3, the mice were divided into two groups: the DMSO-treated group (5% DMSO in saline, 500 μL/mouse, intraperitoneally, twice weekly, four times in total) and the Bortezomib-treated group (1 mg/kg, 500 μL/mouse, intraperitoneally, twice weekly, four times in total) or GC-treated group (only in the #1074 ODX model, Gemcitabine 100 mg/kg, twice a week, 4 times in total, Cisplatin 4 mg/kg, once a week, twice in total) (n = 6 for each). Tumor volume was calculated using the following formula: 0.5 × length × width2. The results compared with the DMSO-treated group were analyzed using a two-way ANOVA with Bonferroni or Tukey’s post hoc test. ns; not significant, * p<0.05, *** p<0.001 HuCCT-1, bile duct cancer cell line; ODX, Organoid-derived xenograft; PDCO, Patient-derived cancer organoid; NSG, NOD; Cg-Prkdcscid Il2rgtm1Wjl/SzJ; DMSO, dimethyl sulfoxide. [file Image1.tiff]

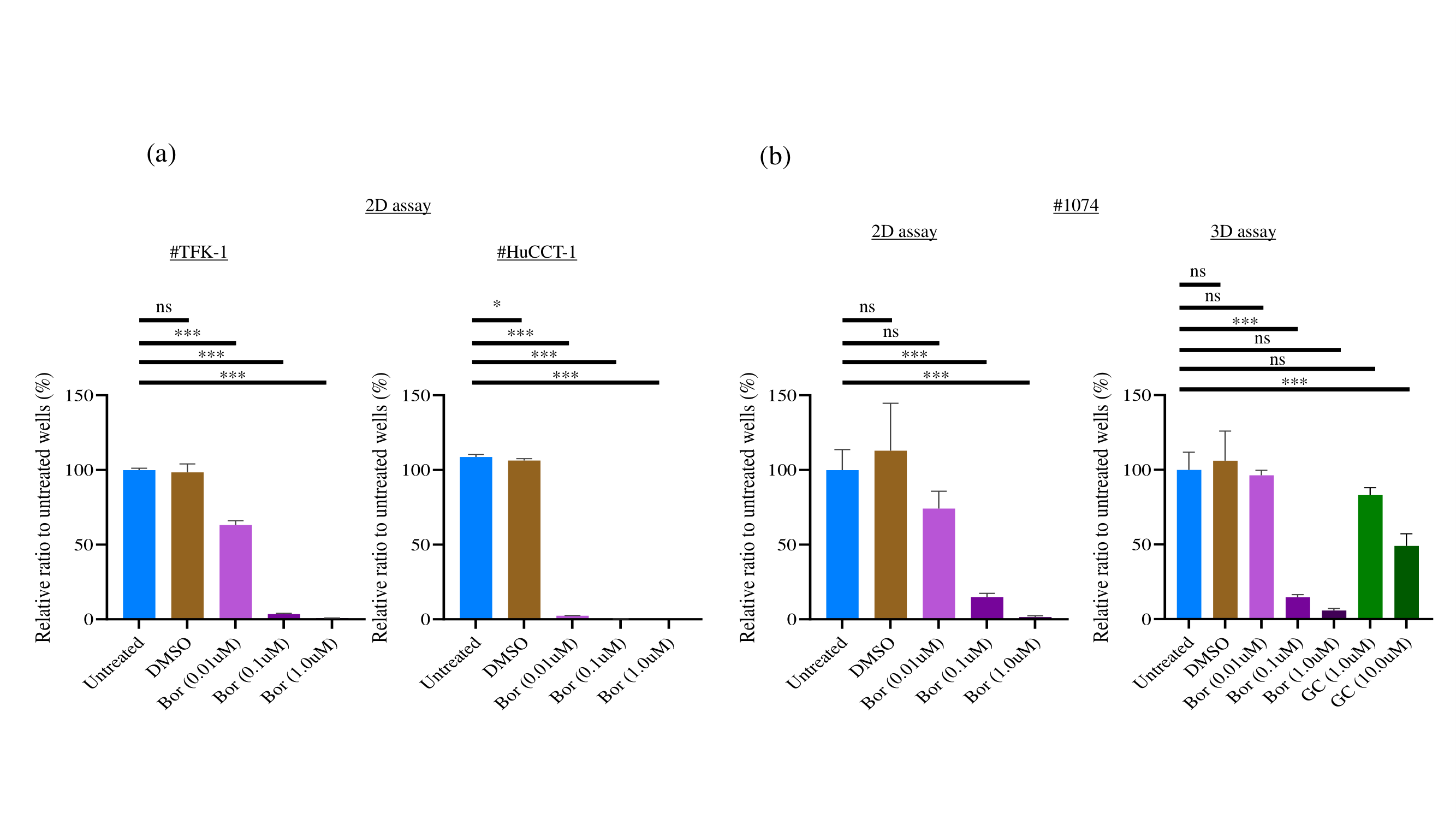

Supplement: Supplementary Figure 2 — Bortezomib showed a significant, strong cell proliferation inhibition toward cholangiocarcinoma cell lines and 2D-cultured PDCOs and 3D ALI PDCOs. (A) TFK-1 and HuCCT-1 cells were seeded at 5000 cells/well in 96-well plates overnight, and compounds were added. After 3 days, the luminescence value was measured using the CellTiter-Glo® Luminescent Cell Viability Assay (Promega, WI, USA). This assay was performed in quadruplicate. (B) For the 2D assay, #1074 PDCOs were seeded at 500 cells/well in 96-well plates with complete organoid culture medium for 3 days, and compounds were added. After 3 days, the luminescence value was measured using the CellTiter-Glo® Luminescent Cell Viability Assay (Promega, WI, USA). This assay was performed in quadruplicate. For the 3D assay, #1045 PDCOs were seeded in a 24-well plate-based ALI organoid culture with a complete organoid culture medium for 3 days; then, the culture medium was replaced with a fresh complete organoid culture medium containing the compounds. After 3 days, the luminescence value of each well was measured using the CellTiter-Glo® 3D Cell Viability Assay (Promega). This assay was performed in triplicate. One-way ANOVA was conducted to evaluate differences among groups, followed by Bonferroni post hoc tests to identify specific group differences. ns; not significant, *p<0.05,*** p<0.001. 2D, two-dimensional; 3D, three-dimensional; ALI, Air-liquid interface; PDCO, Patient-derived cancer organoid. [file Image2.tiff]

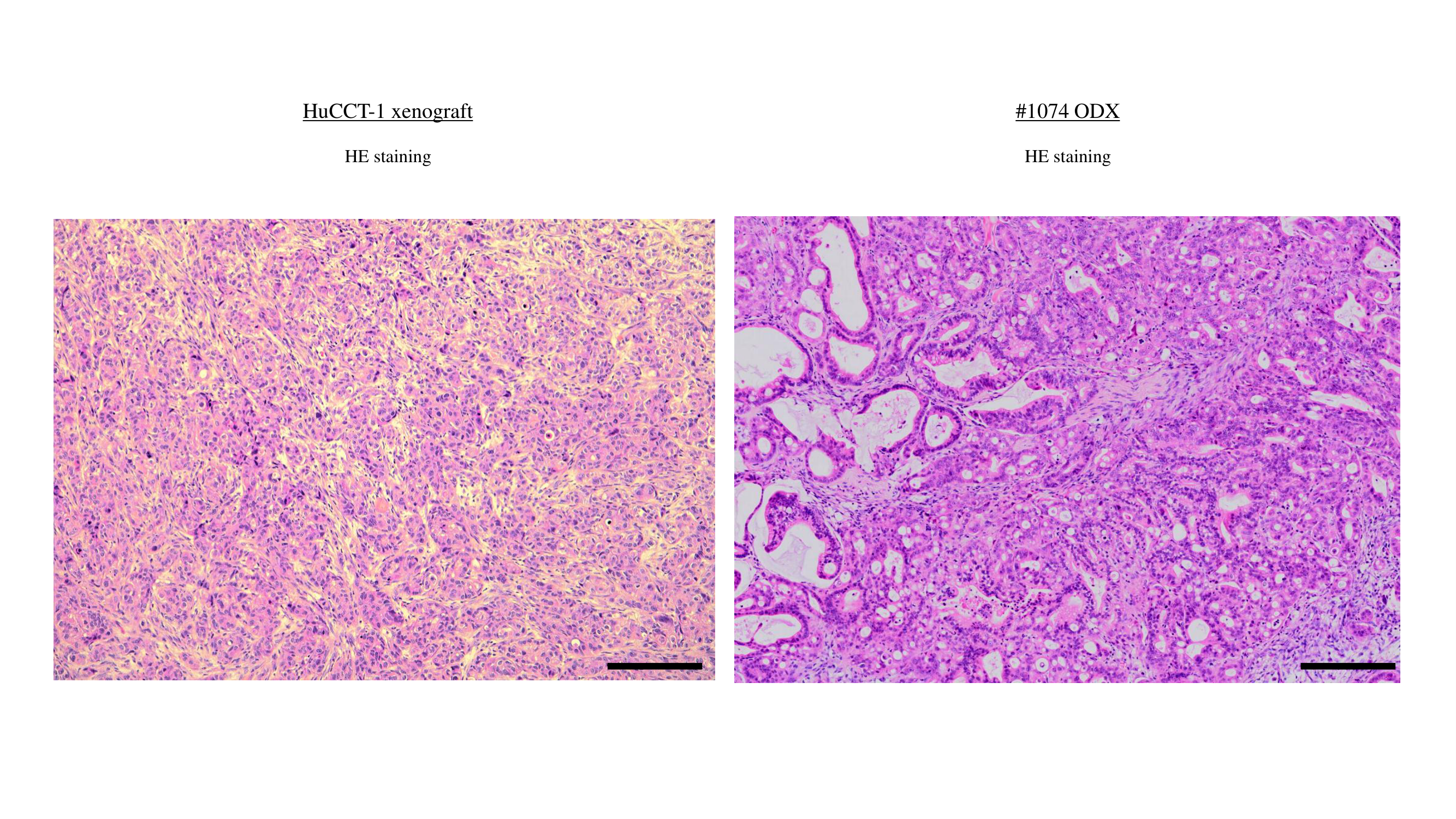

Supplement: Supplementary Figure 3 — H&E staining in BDC cell line (HuCCT-1)-derived xenograft and ODX2 (#1074) Scale bar-200 μm. ODX, Organoid-derived xenograft; H&E, hematoxylin and eosin; BDC, bile duct cancer. [file Image3.tiff]
